# Association of treatment procedures and resilience to symptom load three-years later in a clinical sample of adolescent psychiatric patients

**DOI:** 10.1186/s12888-021-03417-6

**Published:** 2021-08-19

**Authors:** Kari Skulstad Gårdvik, Marite Rygg, Terje Torgersen, Jan Lance Wallander, Stian Lydersen, Marit Sæbø Indredavik

**Affiliations:** 1 Regional Centre for Child and Youth Mental Health and Child Welfare, Department of Mental Health, Faculty of Medicine and Health Sciences, Norwegian University of Science and Technology, Trondheim, Norway; 2 Department of Children and Youth, Clinic of Mental Health Care, St. Olavs hospital, Trondheim University Hospital, Trondheim, Norway; 3 Department of Clinical and Molecular Medicine (IKOM), Faculty of Medicine and Health Sciences, Norwegian University of Science and Technology, Trondheim, Norway; 4 Department of Pediatrics, St. Olavs hospital, Trondheim University Hospital, Trondheim, Norway; 5 Orkdal District Psychiatric Centre, Clinic of Mental Health Care, St. Olavs hospital, Trondheim University Hospital, Trondheim, Norway; 6 Department of Mental Health, Faculty of Medicine and Health Sciences, Norwegian University of Science and Technology, Trondheim, Norway; 7 Psychological Sciences and Health Sciences Research Institute, University of California, Merced, USA

**Keywords:** Mental disorders, Adolescent, Treatment, Resilience, Symptom load

## Abstract

**Background:**

We aimed to examine symptom load in a clinical adolescent population at three-year follow-up and explore associations with standard care treatment procedures and resilience factors upon first presenting at Child and Adolescent Mental Health Services.

**Methods:**

This study is part of a prospective longitudinal cohort study: The Health Survey in Department of Children and Youth, St. Olavs hospital, Norway. A clinical population of 717 (43.5% of eligible) adolescents aged 13–18 years participated in the first study visit (T_1_, 2009–2011). Of these, 447 adolescents with psychiatric disorders, with treatment history from medical records and self-reported resilience factors (Resilience Scale for Adolescents; READ) at T_1_, reported symptom load (Achenbach System of Empirically Based Assessment - Youth Self Report; YSR) three years later aged 16–21 years (T_2_).

**Result:**

At T_1_, 93.0% received individual treatment. The frequency of psychotherapy and medication varied by disorder group and between genders. Overall, psychotherapy was more frequent among girls, whereas medication was more common among boys. Total READ mean value (overall 3.5, SD 0.8), ranged from patients with mood disorders (3.0, SD 0.7) to patients with Attention Deficit Hyperactivity disorder (3.7, SD 0.7), and was lower for girls than boys in all diagnostic groups. At T_2_, the YSR Total Problem mean T-score ranged across the diagnostic groups (48.7, SD 24.0 to 62.7, SD 30.2), with highest symptom scores for those with mood disorders at T_1_, of whom 48.6% had T-scores in the borderline/clinical range (≥60) three years later. Number of psychotherapy sessions was positively associated and Total READ score was negatively associated with the YSR Total Problems T-score (regression coefficient β = 0.5, CI (0.3 to 0.7), *p* < 0.001 and β = − 15.7, CI (− 19.2 to − 12.1), p < 0.001, respectively). The subscale Personal Competence was associated with the lowest Total Problem score for both genders.

**Conclusions:**

Self-reported symptom load was substantial after three years, despite comprehensive treatment procedures. Higher self-reported resilience characteristics were associated with lower symptom load after three years. These results highlight the burden of adolescent psychiatric disorders, the need for extensive interventions and the importance of resilience factors for a positive outcome.

**Supplementary Information:**

The online version contains supplementary material available at 10.1186/s12888-021-03417-6.

## Background

In the transition from adolescence to adulthood, there is an expansion in overall rates of psychiatric disorders [1, 2]. Frequently occurring psychiatric disorders in adolescence are often precursors and strong predictors of comparable disorders in young adulthood [1, 3]. The high degree of continuity of psychopathology from adolescence into young adulthood indicates that the perceived symptom load may be substantial [3, 4]. According to a Lancet report in 2011, psychiatric disorders are the most prominent reason for the global burden of disease in young people [5]. Targeted treatment of psychiatric disorders among adolescents is therefore crucial, and treatment outcomes of standard clinical care is consequently of great interest.

Psychotherapy is often recommended as the first choice of treatment for young people suffering from specific psychiatric disorders. A multilevel meta-analysis synthesizing five decades of cumulative knowledge on effects of youth psychotherapy, states that the impact of therapy differs markedly by target problem, showing larger treatment effects for anxiety than for other problems, and most disappointing effects for depression [6]. As an example of a psychotherapy method widely used for adolescents, cognitive behavioral therapy (CBT) has been a recommended treatment for anxiety disorders, with many studies showing positive effect [7, 8]. However, a Cochrane review from 2020 concluded that CBT was no more effective than non-CBT active control treatments or treatment as usual [9]. Results from the Child/Adolescent Anxiety Multimodal Extended Long-Term Study (CAMELS) found that treatment type was not associated with remission status across the follow-up [10]. Likewise, even though many new treatment methods have been developed for depression during the past decades, their effectiveness has not improved over time [11], according to a meta-analysis of 13-year follow-up of psychotherapy effects on youth depression [12].

As comorbidity of psychiatric disorders is frequent in adolescence [13, 14], especially in clinical samples [15], treatment often needs to involve compound procedures. Also, severe disorders require comprehensive treatment interventions [16–18]. Some transdiagnostic psychotherapy methods have been developed designed to address symptoms of different diagnostic clusters [19, 20]. These have been found to exceed effects of standard manualized treatments with clinically referred youths [21]. The medications for psychiatric disorders are in principle the same for children and adolescents as for adults, but with stricter guidelines. Attention Deficit Hyperactivity disorder (ADHD) is the most common disorder for which medication is recommended [22–24], showing good efficacy and tolerability for children and adolescents [25]. Moreover, antidepressants are often used for mood and anxiety disorders, with selective serotonin reuptake inhibitors (SSRI) as the preferred treatment for children and adolescents [26]. The differences in effect between psychotherapy and antidepressant medication have been found to be small to non-existent in the treatment of adult depression and anxiety disorders [27]. A combination of psychotherapy and pharmacotherapy is the treatment of choice for patients with adult depression [16, 28], but the evidence is limited for children and adolescents [29, 30].

Resilience factors may have implications for the course of treatment, as previous research has found that patients with higher baseline resilience scores, showed less severe psychiatric symptoms after psychotherapeutic interventions [31–33]. Resilience can be referred to as positive adaptation to risk exposure [34] and a more positive psychological outcome than would be expected in case of high levels of environmental adversities [35]. Factors that promote resilience may be categorized into positive individual factors, such as personal and social competence, and may include cognitive factors such as intelligence, personal skills, temperament, and self-esteem [36–38]. Resilience factors can also be contributed at the familial and external social levels, such as family cohesion and support, and social resources and supportive environment outside the family [36–38]. These factors may affect developmental courses of psychiatric disorders and contribute to a better outcome [39, 40]. As previously found in a group of youth with ADHD in the present clinical population, personal resilience characteristics were associated with better psychosocial functioning and less depression and anxiety [41]. In another study of adolescents, higher resilience scores predicted lower scores on levels of depression, anxiety, and obsessive-compulsive symptoms [42], and optimal outcomes of child and adolescent psychiatric disorders are predicted by a combination of personal characteristics and environmental support [43].

Resilience factors may differ between girls and boys in adolescence. Boys compared to girls have reported higher personal competence [36, 38, 44, 45] and social competence [38, 40], whereas girls have reported more access to social resources, which includes supportive family and friends [36, 38, 45]. Furthermore, boys have scored higher on perceived family cohesion than girls [38, 40, 44, 45]. These studies have investigated gender differences in resilience factors in the general population.

The motivation for the present study was to advance knowledge on the progress of psychiatric symptoms in a clinical adolescent population who had received standard care either in out- or inpatient setting in the Child and Adolescent Mental Health Services (CAMHS). Earlier research on the course of symptoms and treatment outcome is mainly conducted on patients with selected psychiatric disorders, recruited to treatment studies. As the impact of therapy differs markedly by target problem [6], research on symptom development must be differentiated by psychiatric disorders. Furthermore, resilience factors may affect psychiatric outcome, but as research on these factors in relation to psychiatric symptoms are primarily carried out in the general population or in specific diagnostic groups, knowledge is scarce about the significance of resilience factors in a general clinical population of adolescents.

The overriding aim of this study was to examine whether psychiatric symptom load three years later was related to the treatment procedures received and resilience factors upon first presenting at mental health clinics for adolescents. We describe characteristics of treatment received in standard adolescent mental health care and symptom load three years later. We hypothesized that symptom load remained substantial and that disorder specific treatment procedures were analogues for girls and boys. Additionally, we hypothesized that having received more psychotherapy sessions or medication was associated with higher symptom load three years later, indicating the large burden of symptoms in this group of former patients. Further, we describe self-reported resilience measures at baseline, specified by psychiatric disorders and gender. We hypothesized that higher resilience factors at baseline was associated with lower symptom load three years later and that boys would report higher resilience factors in personal and social competence domains, whereas girls would report higher social resources.

## Method

### Study design

The study is part of the Health Survey in Department of Children and Youth, Clinic of Mental Health Care, St. Olav’s hospital, Trondheim University Hospital, Norway (St. Olav CAP Survey), a prospective longitudinal cohort study of a defined clinical population assessed at two time points. At time point 1 (T_1_) (2009–2011), all patients aged 13–18 years who visited the Department of Children and Youth at least once over a 2-year period were invited at their first attendance. The exclusion criteria were difficulties in answering the survey due to insufficient language skills, low cognitive function, visual impairments, or unstable psychiatric state. Emergency patients were invited to take part once they entered a stable phase. The study design is detailed in a previous publication [15]. The participants and their parents received standard application of mental health services. At 3-year follow-up (T_2_) (2012–2014), age 16–21 years, data were collected from the T_1_ enrolled sample and their parents, by an electronic survey and a diagnostic telephone interview performed by trained professionals.

### Participants

**Fig. 1:**
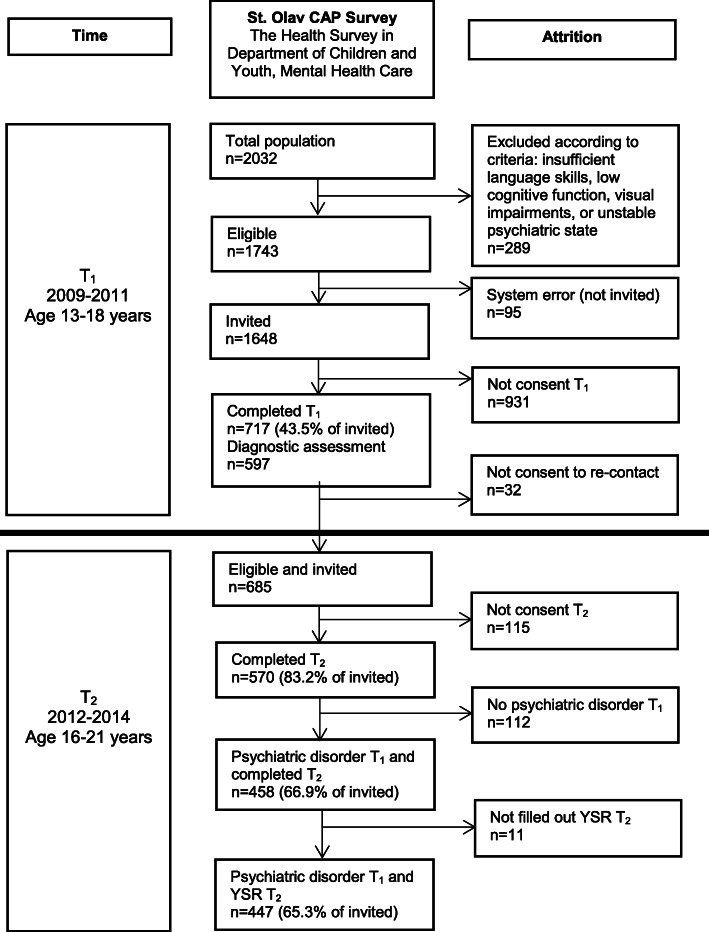
Flow-chart of the recruitment and attrition in the present study

In the T_1_ study period, 2032 adolescent patients had at least one attendance in the Department of Children and Youth. Figure 1 illustrates the participant flow in each stage of the survey. Among the possible participants in the study period (*n* = 2032), *n* = 289 were excluded, and *n* = 1743 were eligible. Since *n* = 95 were lost to registration (missing), *n* = 1648 (81.1%) were invited. Of these, *n* = 717 (43.5%) participated (393, 54.8% girls), and *n* = 931 (56.5%) declined or did not respond to the invitation. The representativeness of the study population at T_1_ has been investigated in a previous publication, including in depth attrition analyses [15]. Of the T_1_ participants, *n* = 597 had completed diagnostic assessment investigating the reason for referral. The number of participants by single-year age-groups were: 13 years: *n* = 79 (17.7%), 14 years: *n* = 87 (19.5%), 15 years: *n* = 80 (17.9%), 16 years: *n* = 83 (18.6%), 17 years: *n* = 82 (18.3%), 18 years: *n* = 36 (8.0%). At T_2_, all T_1_ participants who previously consented to further inquiry were invited (eligible *n* = 685), and 570 (83% of eligible) completed the follow-up questionnaire (324, 56.8% girls). The present study included the 447 (65.3% of invited) participants who had a psychiatric disorder at T_1_ and had filled out YSR at T_2_ (254, 56.8% girls). Comparing participants versus non-participants at T_2_, the proportion of girls was higher among participants, while age and socioeconomic status were similar.

### Measures

**Psychiatric Diagnoses** at T_1_ were set in ordinary clinical practice according to the International Statistical Classification of Disease and Related Health Problems (ICD-10) multiaxial diagnostics (axes I-VI) [46]. The diagnostic process followed standardized procedures for assessment and diagnosis of common adolescent psychiatric disorders, depending upon a comprehensive developmental history and interviews with the adolescents and their parents. The semi-structured Schedule for Affective Disorders and Schizophrenia for School-Age Children (K-SADS) [47] was used in some cases, and in others The Development And Well-Being Assessment (DAWBA) [48] and diverse rating scales appropriate for the presenting problem were used. The diagnoses were set by a clinical psychologist or a child and adolescent psychiatrist, both of which are qualified to set diagnoses in CAMHS in Norway, based on all accessible clinical information, after consensus discussion with other professionals from the multi-disciplinary team. Somatic examinations were added to the assessments if indicated, and possible coexisting disorders were investigated.

In the present study, disorders were grouped into the following categories, based on ICD-10 diagnoses at T_1_; (1) Any psychiatric disorder, (2) Anxiety disorders (ICD-codes F40-F44, F48, F93), (3) Mood disorders (ICD-codes F31-F34, F38, F39), (4) ADHD (ICD-code F90) and (5) Other (ICD-codes F10-F19, F20-F21, F28-F29, F50, F54, F59-F60, F84, F91-F92, F94-F95, F98). Due to few participants in some diagnostic groups, for example autism and eating disorders, we chose to merge these diagnoses into one larger group of “other psychiatric disorders”.

**Treatment** given at T_1_ was registered in medical records by type (cognitive, neurobiological, psychodynamic, psychoeducational, social-relational, medication), participant (individual, group, parent, family), number of sessions, duration of treatment, in-patient or out-patient, indirect patient work by counselling municipal services, giving consultations to service agencies already engaged with the patient. In this study, we classified treatment procedures into psychotherapy (specified or unspecified, and divided into numbers of sessions given; < 10, 10–30, > 30), medication according to Anatomical Therapeutic Chemical (ATC) codes, counselling parents/family therapy and counselling municipal services, all classified as present or not (Yes/No). Treatment were provided according to guidelines for specific diagnosis.

**Resilience** factors were measured at T_1_ using the Resilience Scale for Adolescents (READ), a self-report questionnaire measuring the ability to manage stress and negative experiences [36]. READ is a 28-item scale with positively formulated items organized in five subscales: Personal Competence, Social Competence, Structured Style, Family Cohesion and Social Resources. READ is based on the Resilience Scale for Adults [49] and was developed in Norway in 2006 with a 5-point Likert-type response scale from 1 = Totally Disagree to 5 = Totally Agree. Higher scores on the READ indicate higher level of resilience factors. The READ scale is widely used in research and has shown good psychometric properties in validation studies [38, 45]. In this study, we used mean item scores for each scale (values between 1 and 5). Internal consistency measured as Cronbach’s alpha for the subscales was .89 (Personal Competence), .84 (Social Competence), .73 (Structured Style), .91 (Family Cohesion), and .84 (Social Resources), which would be generally regarded in the range from acceptable to excellent [50].

**Psychiatric symptom load** at T_2_ was investigated using the Achenbach System of Empirically Based Assessment – Youth Self Report (YSR) [51]. This is a screening instrument for emotional and behavioral symptoms, designed to assess a broad array of psychopathological manifestations in adolescents, consisting of both a competence scale and a problem scale. For the purpose of this study, the latter was used, consisting of 103 problem items, rated on a 3-point scale (0 = not true; 1 = somewhat or sometimes true; 2 = very true or often true), during the past six months. In this study, Total problems T-score was used as the measure of symptom load at T_2_, with cut-off at scores ≥60 as borderline/clinical range, and < 60 as normal range, as recommended in the manual [51]. The scale has shown good psychometric properties and is widely used in research and clinical services in different populations [52, 53].

**Socioeconomic Status (SES)** was measured at T_1_ by the mothers’ highest level of education, categorized in eight levels: (1) less than 9-year primary school; (2) completed 9-year primary school; (3) one or two years in high school; (4) completed high school; (5) completed high school and one-year education/training after high school; (6) academy/university for up to and including four years; (7) academy/university for five years or more; (8) academy/university including PhD.

### Statistical analyses

In this study, distributions were checked for normality using Q-Q Plots. Confidence intervals and tests for differences in age, SES, symptom load and resilience measures between girls and boys were based on Student’s t-test for independent samples. We compared proportions of treatment measures between girls and boys by using the Newcombe hybrid score confidence intervals, as recommended [54], and the Pearson Chi squared test. Linear regression was used with symptom load at T_2_ as dependent variable and resilience and treatment procedures reported at T_1_ as covariates, one at a time, to study their associations. These regression analyses were accomplished adjusted for age at T_1_ and SES as possible confounders. We have reported 95% confidence intervals (CI) where relevant, and two-sided *p*-values < 0.05 were considered statistically significant. However, due to testing multiple hypotheses and thus the possibility of Type I error, p-values between 0.01 and 0.05 should be interpreted critically. The Newcombe CI were calculated in Stata 16, and the other calculations in SPSS 27.

### Ethics

Written informed consent was obtained from adolescents and parents prior to inclusion at T_1_, and from the adolescents at T_2_, according to study procedures. The Norwegian Social Science Data Services, The Data Protection Official for Research, gave permission to investigate the representativeness of the study at T_1_ (reference number: 19976). Study approval was given by the Regional committee for Medical and Health Research Ethics of Central Norway (reference numbers CAP survey T_1_: 4.2008.1393, T_2_: 2011/1435/REK Midt, and present study using T_1_ and T_2_ data: 2017/589/REK Midt).

## Results

### Descriptive information

The 447 participants had mean age at T_1_: 15.7 years (SD 1.7) and T_2_: 18.5 years (SD 1.6). Girls were significantly older than boys at both time points (16.0 years (SD 1.7) vs 15.3 years (SD 1.6), *p* < 0.001, and 19.0 years (SD 1.7) vs 18.3 years (SD 1.6), *p* < 0.001, respectively). SES was measured at T_1_ (*n* = 327/447): Mean 4.9 (SD 1.7), for girls (*n* = 181/254) 4.9 (SD 1.7) and boys (*n* = 146/193) 4.8 (SD 1.7). At T_1_, ADHD was the most frequent diagnostic group (46.3%) in the total sample, followed by anxiety disorder (33.8%) and mood disorder (23.9%), when both primary and additional diagnoses were included. Anxiety disorder (40.5%) was the most frequent diagnostic group among girls, and ADHD (62.2%) was among boys (Table 1). Comorbid psychiatric disorders were found among 30.2% of the participants, with no gender differences (data not shown).

**Table 1:** Outpatient treatment procedures at T_1_ differentiated by psychiatric disorders, including comorbid disorders at T_1_, overall and separately for girls and boys

			Outpatient treatment procedures T_**1**_													
			Individual treatment procedures										Counselling			
			Any individual		Psychotherapy^b^						Medication^c^		Parents/		Municipal	
			treatment										family therapy		services	
Psychiatric disorders^a^ T_**1**_					< 10 sessions		10–30 sessions		> 30 sessions							
	n	(%)	n	(%)	n	(%)	n	(%)	n	(%)	n	(%)	n	(%)	n	(%)
**Total sample**																
Any psych disorder	447		414/445	(93.0)	177/424	(41.7)	120/424	(28.3)	53/424	(12.5)	236/445	(53.0)	295/438	(67.4)	214/436	(49.1)
Anxiety disorders	151/447	(33.8)	139/150	(92.7)	46/142	(32.4)	58/142	(40.8)	28/142	(19.7)	56/150	(37.3)	114/149	(76.5)	76/149	(51.0)
Mood disorders	107/447	(23.9)	99/106	(93.4)	28/101	(27.7)	45/101	(44.6)	20/101	(19.8)	52/106	(49.1)	71/105	(67.6)	42/105	(40.0)
ADHD	207/447	(46.3)	199/207	(96.1)	99/196	(50.5)	35/196	(17.9)	14/196	(7.1)	168/207	(81.2)	131/203	(64.5)	116/203	(57.1)
Other psych disorders	99/447	(22.1)	89/99	(89.9)	35/93	(37.6)	28/93	(30.1)	15/93	(16.1)	46/99	(46.5)	59/97	(60.8)	48/95	(50.5)
**Girls**																
Any psych disorder	254		239/253	(94.5)	88/242	(36.4)	87/242	(36.0)	44/242	(18.2)	114/253	(45.1)	172/250	(68.8)	117/248	(47.2)
Anxiety disorders	103/254	(40.5)	96/102	(94.1)	28/96	(29.2)	41/96	(42.7)	23/96	(24.0)	36/102	(35.3)	76/101	(75.3)	50/101	(49.5)
Mood disorders	88/254	(34.6)	83/88	(94.3)	21/83	(25.3)	39/83	(47.0)	18/83	(21.7)	44/88	(50.0)	58/87	(66.7)	33/87	(37.9)
ADHD	87/254	(34.3)	84/87	(96.5)	40/84	(47.6)	20/84	(23.8)	13/84	(15.5)	66/87	(75.9)	60/87	(69.0)	54/87	(62.1)
Other psych disorders	49/254	(19.3)	45/49	(91.8)	13/44	(29.6)	18/44	(40.9)	11/44	(25.0)	16/49	(32.7)	28/47	(59.6)	20/45	(44.4)
**Boys**																
Any psych disorder	193		175/192	(91.1)	89/182	(48.9)	33/182	(18.1)	9/182	(5.0)	122/192	(63.5)	123/188	(65.4)	97/188	(51.6)
Anxiety disorders	48/193	(24.9)	43/48	(89.6)	18/46	(39.1)	17/46	(37.0)	5/46	(10.9)	20/48	(41.7)	38/48	(79.2)	26/48	(54.2)
Mood disorders	19/193	(9.8)	16/18	(88.9)	7/18	(38.9)	6/18	(33.3)	< 5/18		8/18	(44.4)	13/18	(72.2)	9/18	(50.0)
ADHD	120/193	(62.2)	115/120	(95.8)	59/112	(52.7)	15/112	(13.4)	< 5/112		102/120	(85.0)	71/116	(61.2)	62/116	(53.4)
Other psych disorders	50/193	(25.9)	44/50	(88.0)	22/49	(44.9)	10/49	(20.4)	< 5/49		30/50	(60.0)	31/50	(62.0)	28/50	(56.0)

Note: ^a^ Psychiatric disorders include both primary and additional diagnoses

^b^ Psychotherapy include both specified and unspecified psychotherapy

^c^ Medication includes medication for psychiatric disorders; according to Anatomical Therapeutic Chemical (ATC) codes Yes/No. Supplementary Material Table S1 shows the medication given for primary diagnoses differentiated by ATC-codes

### Treatment procedures

Frequency of different treatment procedures are presented in Table 1. In the total sample, 93.0% received individual treatment. The frequency of psychotherapy sessions varied by disorder group: Among patients with ADHD, 50.5% received less than 10 sessions, while patients with mood disorders and anxiety disorders received the highest number of sessions; 19.8 and 19.7% respectively received more than 30 sessions. Medication was most frequent in the ADHD group (81.2%). The rates of parent counselling or family therapy were between 60.8 and 76.5% in the total sample, with the highest rate for anxiety disorders, with no difference between genders. Counselling municipal services was provided for 49.1% in the total sample.

Gender comparisons in treatment procedures are shown in in Table S2. Psychotherapy was more frequent among girls overall (RD = 18.9, CI (11.2 to 26.4), *p* < 0.001), as well as in all groups of psychiatric disorders, with the largest gender difference occurring in the group of other psychiatric disorders. Medication was significantly less common for girls versus boys overall (RD = − 18.4, CI (− 27.3 to − 9.1), *p* < 0.001) and in the group of other psychiatric disorders. There was no gender difference for ADHD medication.

### Resilience factors

As shown in Table 2, Total READ mean value was 3.5 (SD 0.8) for patients with any psychiatric disorder, ranging from 3.0 (SD 0.7) for patients with mood disorders to 3.7 (SD 0.7) for patients with ADHD. Girls had lower total READ mean values than boys for any disorder and for all disorder groups except for mood disorders. The subscale Personal Competence showed the largest gender differences, with statistically significantly higher mean values for boys than girls, in all diagnostic groups (Table S3).

**Table 2:** Resilience scales at T_1_ differentiated by psychiatric disorder groups, overall and separately for girls and boys

Psychiatric disorders^a^ T_**1**_			Personal competence		Social competence		Structured style		Family cohesion		Social resources		Total READ		Total READ		
															Girls versus Boys		
	n	(%)	Mean	(SD)	Mean	(SD)	Mean	(SD)	Mean	(SD)	Mean	(SD)	Mean	(SD)	Diff.	95% CI^b^	***p***-value^b^
**Total sample**																	
Any psychiatric disorder	447		3.3	(1.0)	3.7	(0.9)	3.1	(0.9)	3.5	(1.0)	4.1	(0.8)	3.5	(0.8)			
Anxiety disorders	151/447	(33.8)	3.1	(0.9)	3.6	(0.9)	3.0	(0.9)	3.5	(1.1)	4.0	(0.9)	3.5	(0.8)			
Mood disorders	107/447	(23.9)	2.5	(0.9)	3.2	(0.9)	2.6	(0.8)	3.0	(0.9)	3.6	(0.9)	3.0	(0.7)			
ADHD	207/447	(46.3)	3.5	(0.9)	3.8	(0.9)	3.2	(0.9)	3.7	(0.9)	4.2	(0.7)	3.7	(0.7)			
Other psychiatric disorders	99/447	(22.1)	3.3	(0.9)	3.7	(0.9)	3.1	(0.9)	3.7	(1.0)	4.2	(0.7)	3.6	(0.7)			
**Girls**																	
Any psychiatric disorder	254		2.9	(0.9)	3,6	(0.9)	2.9	(0.9)	3.4	(1.1)	4.0	(0.9)	3.3	(0.7)	−0.5	−0.6 to −0.3	< 0.001
Anxiety disorders	103/254	(40.5)	2.9	(0.9)	3.5	(0.9)	2.9	(0.9)	3.3	(1.1)	4.0	(0.9)	3.3	(0.8)	−0.5	−0.7 to −0.2	< 0.001
Mood disorders	88/254	(34.6)	2.4	(0.8)	3.2	(0.8)	2.6	(0.7)	3.0	(0.9)	3.6	(0.9)	2.9	(0.6)	−0.3	−0.6 to 0.0	0.080
ADHD	87/254	(34.3)	3.1	(1.0)	3.7	(1.0)	2.8	(0.9)	3.5	(1.0)	4.1	(0.8)	3.4	(0.7)	−0.5	−0.7 to − 0.3	< 0.001
Other psychiatric disorders	49/254	(19.3)	2.9	(0.7)	3.6	(0.9)	2.9	(0.9)	3.5	(1.1)	4.1	(0.7)	3.4	(0.7)	−0.4	− 0.6 to − 0.1	0.013
**Boys**																	
Any psychiatric disorder	193		3.7	(0.8)	3.9	(0.9)	3.4	(0.9)	3.8	(0.9)	4.3	(0.7)	3.8	(0.7)			
Anxiety disorders	48/193	(24.9)	3.6	(0.7)	4.0	(0.8)	3.3	(0.8)	3.8	(0.8)	4.3	(0.7)	3.8	(0.6)			
Mood disorders	19/193	(9.8)	3.0	(0.9)	3.4	(1.0)	2.8	(0.9)	3.2	(1.1)	3.9	(1.0)	3.2	(0.8)			
ADHD	120/193	(62.2)	3.8	(0.8)	4.0	(0.9)	3.5	(0.9)	3.9	(0.8)	4.3	(0.7)	3.9	(0.7)			
Other psychiatric disorders	50/193	(25.9)	3.7	(0.8)	3.8	(1.0)	3.3	(0.9)	3.8	(0.9)	4.2	(0.7)	3.8	(0.7)			

Note: Resilience measures using READ = Resilience Scale for Adolescents, based on a 5-point Likert scale (1 = Totally Disagree to 5 = Totally Agree, higher scores indicate higher level of resilience factors), SD = Standard Deviation, CI = Confidence Interval

^a^ Psychiatric disorders include both primary and additional diagnoses

^b^ Confidence intervals and tests for differences between girls and boys were based on Student’s t-test for independent samples

### Psychiatric symptom load after three years

In the total sample, the YSR Total Problem mean T-score at T_2_ ranged from 48.6 (SD 26.3) to 62.7 (SD 28.0) across the diagnostic groups. The highest symptom scores were for those with mood disorders at T_1_, of whom 48.6% had T-scores in the borderline/clinical range (≥60) three years later (Table 3). Comparing the T-scores for participants with and without a diagnosis at T_2_ (n_diagnosis_ = 314, n_no diagnosis_ = 108), the mean T-scores were 55.5 (SD 26.8) versus 34.0 (SD 18.3), respectively. The YSR scores were significantly higher among girls than among boys in all diagnostic groups, especially in the groups of mood disorders and other psychiatric disorders (Mean difference 24.8, CI (6.7 to 11.6), *p* < 0.001 and 25.2, CI (14.2 to 36.2), *p* < 0.001, respectively) (Table 3). The gender differences were present when comparing the T-scores for participants with or without a diagnosis at T_2_, with girls (n_diagnosis_ = 182, n_no diagnosis_ = 57) having mean T-scores of 64.5 (SD 26.4) versus 38.6 (SD 18.2), and boys (n_diagnosis_ = 132, n_no diagnosis_ = 51) mean T-scores 43.1 (SD 21.9) versus 28.7 (SD 17.0), respectively.

**Table 3:** Symptom load at T_2_ differentiated by psychiatric disorders at T_1_, overall and separately for girls and boys

	YSR Total Problem T-Score at T_**2**_								
Psychiatric disorders^a^ T_**1**_			≥ 60^b^				Girls versus Boys		
	n	(%)	n	(%)	Mean	(SD)	Mean difference	95% CI^c^	p-value^c^
**Total sample**									
Any psychiatric disorder	447		137/447	(30.6)	50.5	(26.5)			
Anxiety disorder	151/447	(33.8)	46/151	(30.5)	50.1	(24.0)			
Mood disorder	107/447	(23.9)	52/107	(48.6)	62.7	(28.0)			
ADHD	207/447	(46.3)	57/207	(27.5)	48.6	(26.3)			
Other psychiatric disorder	99/447	(22.1)	33/99	(33.3)	52.9	(30.2)			
**Girls**									
Any psychiatric disorder	254		103/254	(40.5)	58.7	(27.0)	18.9	14.3 to 23.4	< 0.001
Anxiety disorder	103/254	(40.5)	37/103	(35.9)	54.1	(23.4)	12.8	4.8 to 20.9	0.002
Mood disorder	88/254	(34.6)	49/88	(55.7)	67.1	(27.1)	24.8	6.7 to 11.6	< 0.001
ADHD	87/254	(34.3)	34/87	(39.1)	59.3	(28.9)	18.6	11.4 to 25.8	< 0.001
Other psychiatric disorder	49/254	(19.3)	24/49	(49.0)	65.6	(29.7)	25.2	14.2 to 36.2	< 0.001
**Boys**									
Any psychiatric disorder	193		34/193	(17.6)	39.8	(21.6)			
Anxiety disorder	48/193	(24.9)	9/48	(18.7)	41.3	(23.2)			
Mood disorder	19/193	(9.8)	3/19	(15.8)	42.3	(22.7)			
ADHD	120/193	(62.2)	23/120	(19.2)	40.8	(21.2)			
Other psychiatric disorder	50/193	(25.9)	9/50	(18.0)	40.4	(25.3)			

Note: Symptom load is measured by using Youth Self Report (YSR, Achenbach System of Empirically Based Assessment), Total Problem T-score, with scores ≥ 60 as borderline and clinical range, and < 60 as normal range. SD = Standard Deviation, CI = Confidence Interval

^a^ Psychiatric disorders include both primary and additional diagnoses

^b^ Borderline/clinical range

^c^ Confidence intervals and tests for differences between girls and boys were based on Student’s t-test for independent samples

### Associations between treatment characteristics and symptom load 3 years later

Older age and lower SES were significantly associated with higher symptom load at 3-year follow-up in the total sample (Age T_2_: regression coefficient β = 2.5, CI (1.1 to 4.0), *p* = 0.001; SES: β = − 2.1, CI (− 3.7 to − 0.5), *p* = 0.012), and for girls only (Age T_2_: β = 2.2, CI (0.2 to 4.2), *p* = 0.033; SES: β = − 2.9, CI (− 5.3 to − 0.6), *p* = 0.014). Linear regression analysis with YSR Total Problem T-score at T_2_ as dependent variable and treatment procedures as covariates were therefore performed adjusted for age and SES.

There was a statistically significant positive association between having received psychotherapy at T_1_ and symptom load three years later for the total sample for any psychiatric disorder (β = 9.9, CI (2.4 to 17.4), *p* = 0.010). When increasing the number of psychotherapy sessions in the total sample by 1 session, the YSR Total Problems T-score increased with 0.5 units (β = 0.5, CI (0.3 to 0.7), *p* < 0.001) (Table 4). This association was present only for participants with a diagnoses at T_2_ (n_diagnosis_ = 314, β = 0.6, CI (0.4 to 0.9), p < 0.001), (n_no diagnosis_ = 108, β = 0.1, CI (− 0.2 to 4.0), *p* = 0.519). The significant associations were found in all diagnostic groups except for mood disorders (Table 4). The significant associations were found for girls with anxiety disorders and ADHD, as well as any psychiatric disorders.

**Table 4:** Linear regression analysis with YSR Total Problems T-score at 3-year follow up as dependent variable and treatment procedures as covariates one at a time, adjusted for age and SES, differentiated by psychiatric disorders

				YSR Total Problems T-score at T_**2**_				
Psychiatric disorders T_**1**_^a^			Treatment procedures	Adjusted for age T_**1**_ and SES				
	n	(%)		n	%	β	95% CI	***p***-value
**Total sample**								
Any psychiatric disorder	447		Psychotherapy^b^	299/424	(70.5)	0.5	0.3 to 0.7	< 0.001
			Medication^c^	236/445	(53.0)	−1.7	−7.4 to 4.0	0.566
Anxiety disorders	151/447	(33.8)	Psychotherapy	95/150	(63.3)	0.5	0.1 to 0.8	0.007
			Medication	56/150	(37.3)	9.0	−0.6 to 18.7	0.067
Mood disorders	107/447	(23.9)	Psychotherapy	60/106	(56.6)	0.3	−0.1 to 0.8	0.169
			Medication	52/106	(49.1)	−2.1	−16.6 to 12.4	0.773
ADHD	207/447	(46.3)	Psychotherapy	146/207	(70.5)	0.7	0.4 to 1.0	< 0.001
			Medication	168/207	(81.2)	−5.7	− 16.1 to 4.6	0.275
Other psychiatric disorder	99/447	(22.1)	Psychotherapy	72/99	(72.7)	0.6	0.1 to 1.0	0.011
			Medication	46/99	(46.5)	−6.9	−20.5 to 6.7	0.313
**Girls**								
Any psychiatric disorder	254		Psychotherapy	165/242	(68.2)	0.4	0.2 to 0.7	0.002
			Medication	114/253	(45.1)	−1.7	−10.0 to 6.6	0.692
Anxiety disorders	103/254	(40.6)	Psychotherapy	59/102	(57.8)	0.6	0.2 to 1.0	0.007
			Medication	36/102	(35.3)	7.4	−5.6 to 20.3	0.260
Mood disorders	88/254	(34.7)	Psychotherapy	51/88	(58.0)	0.2	−0.3 to −0.7	0.414
			Medication	44/88	(50.0)	0.4	−14.7 to 15.4	0.960
ADHD	87/254	(34.3)	Psychotherapy	62/87	(71.3)	0.7	0.3 to 1.2	0.002
			Medication	66/87	(75.9)	−9.6	−25.4 to 6.1	0.226
Other psychiatric disorder	49/254	(19.3)	Psychotherapy	36/49	(73.5)	0.5	−0.1 to 1.0	0.103
			Medication	16/49	(32.7)	12.0	−11.3 to 35.3	0.304
**Boys**								
Any psychiatric disorder	193		Psychotherapy	134/182	(73.6)	−0.1	−0.4 to 0.3	0.780
			Medication	122/192	(63.5)	7.4	0.7 to 14.2	0.032
Anxiety disorders	48/193	(24.9)	Psychotherapy	36/48	(75.0)	−0.2	−0.9 to 0.4	0.451
			Medication	20/48	(41.7)	12.9	−0.5 to 26.3	0.059
Mood disorders	19/193	(9.8)	Psychotherapy	9/18	(50.0)	−0.9	−3.2 to 0.4	0.141
			Medication	8/18	(44.4)	−9.8	−34.0 to 14.5	0.347
ADHD	120/193	(62.2)	Psychotherapy	84/120	(70.0)	0.0	−0.5 to 0.6	0.972
			Medication	102/120	(85.0)	5.7	−7.3 to 18.7	0.385
Other psychiatric disorder	50/193	(25.9)	Psychotherapy	36/50	(72.0)	−0.1	−0.9 to 0.6	0.767
			Medication	30/50	(60.0)	−0.9	−16.7 to 15.0	0.913

Note: Symptom load is measured by using Youth Self Report (YSR, Achenbach System of Empirically Based Assessment), Total Problem T-score, SES = Socioeconomic Status measured by level of mothers’ education (1 = lowest level of education, 9 = highest level of education), β = Regression Coefficient, CI = Confidence Interval

^a^ Psychiatric disorders include both primary and additional diagnoses

^b^ Psychotherapy by number of sessions: 1 session as the measurement unit

^c^ Medication includes medication for psychiatric disorders; according to Anatomical Therapeutic Chemical (ATC) codes Yes/No

Medication prescribed at T_1_ was not statistical significantly associated with symptom load three years later for the total sample (Table 4). For boys only, medication was associated with an increased YSR Total Problem T-score of over 7 at follow-up for any psychiatric disorder (β = 7.4, CI (0.7 to 14.2), *p* = 0.032), but no statistically significant associations were found when specifying by psychiatric disorders. No statistically significant associations were found between counselling parents or counselling municipal services and symptom load at follow-up (data not shown).

### Associations between resilience factors and symptom load 3 years later

Linear regression analysis with YSR Total Problem T-score at 3-year follow up as dependent variable and READ resilience scale scores as covariates showed significant negative associations for Total READ and for all subscale scores, adjusted for age and SES (Table 5). Increasing the Total READ score by 1 unit (scale 1–5), the YSR Total Problems T-score decreased by 15.7 units (β = − 15.7, CI (− 19.2 to − 12.1), *p* < 0.001). Associations were present both for participants with and without a diagnosis at T_2_ (n_diagnosis_ = 226, Total READ β = − 17.9, CI (− 22.1 to − 13.7), p < 0.001), (n_no diagnosis_ = 84, Total READ β = − 7.1, CI (− 12.3 to − 1.9), *p* = 0.008). READ Personal Competence was the subscale associated with the largest decrease in Total Problem score for both genders (girls: β = − 11.8, CI (− 15.9 to − 7.6), p < 0.001 and boys: β = − 9.4, CI (− 13.5 to − 5.2), p < 0.001) (Table 5). Linear regression analysis including the five READ subscales simultaneously, showed that Personal Competence and Family Cohesion remained associated with a decrease in Total Problem score, but for girls only (Table S4). When differentiating between psychiatric disorder groups, linear regression analysis with YSR Total Problem T-score as dependent variable and Total READ scale as covariate showed significant negative associations for Total READ in all disorder groups, except for boys with mood disorders (Table S5).

**Table 5:** Linear regression analysis with YSR Total Problems T-score at 3-year follow up as dependent variable and resilience factors as covariates one at a time, adjusted for age and SES, overall and separately for girls and boys

		YSR Total Problems T-score at T_**2**_		
Resilience measures		Adjusted for age T_**1**_ and SES		
	n	β	95% CI	***p***-value
**Total sample**	447			
Total resilience	444/447	−15.7	−19.2 to − 12.1	< 0.001
Personal competence	446/447	−13.0	− 15.7 to − 10.2	< 0.001
Social competence	444/447	−7.0	− 10.1 to −4.0	< 0.001
Structured style	445/447	−11.0	−14.0 to −8.0	< 0.001
Family cohesion	444/447	−10.5	− 13.3 to −7.8	< 0.001
Social resources	444/447	−10.3	−13.6 to −6.9	< 0.001
**Girls**	254			
Total resilience	253/254	−14.7	−19.8 to −9.6	< 0.001
Personal competence	254/254	−11.8	−15.9 to −7.6	< 0.001
Social competence	253/254	−5.1	−9.5 to −0.7	0.022
Structured style	254/254	−10.3	−14.9 to −5.7	< 0.001
Family cohesion	253/254	−10.5	−14.1 to −7.0	< 0.001
Social resources	253/254	−9.2	−13.6 to −4.9	< 0.001
**Boys**	193			
Total resilience	191/193	−10.8	−15.7 to −6.0	< 0.001
Personal competence	192/193	−9.4	−13.5 to −5.2	< 0.001
Social competence	191/193	−6.1	−9.9 to −2.3	0.002
Structured style	191/193	−7.4	−11.2 to −3.6	< 0.001
Family cohesion	191/193	−6.6	−10.7 to −2.5	0.002
Social resources	191/193	−7.7	−12.6 to − 2.9	0.002

Note: Symptom load is measured by using Youth Self Report (YSR, Achenbach System of Empirically Based Assessment), Total Problem T-score, Resilience measures using READ = Resilience Scale for Adolescents, based on a 5-point Likert scale (1 = Totally Disagree, 5 = Totally Agree, higher scores indicate higher level of resilience factors), SES = Socioeconomic Status measured by level of mothers education (1 = lowest level of education, 9 = highest level of education), β = Regression Coefficient, CI = Confidence Interval

## Discussion

This is one of few longitudinal surveys studying the potential impact of standard care and resilience factors on subsequent symptom level in a general clinical psychiatric outpatient population of adolescents. The symptom load three years after referral was substantial, where one out of three reported symptoms that places them in the borderline/clinical range. Differentiated by psychiatric disorders, the former patients with ADHD reported the lowest symptom load, whereas those with mood disorders, especially girls, reported the highest symptom load. One main finding was that patients with mood disorders, and especially girls, had received the highest number of psychotherapy sessions, and yet had the highest symptom load after three years. One out of five patients with mood as well as with an anxiety disorder received more than 30 psychotherapy sessions. In contrast, patients with ADHD, and especially boys, received the fewest psychotherapy sessions and had the largest rate of medication as their treatment. Medication given at baseline was marginally associated with higher symptom scores after three years for boys only. Resilience factors were reported to be lowest among patients with mood disorders and highest among ADHD patients. In all diagnostic groups, self-reported resilience factors were lower among girls than boys. Reporting higher resilience factors was associated with lower symptom load after three years, suggesting a protective potential for personal resources.

Our findings of a considerable symptom load three years after referral were similar to the reported symptom load in other studies of outpatient child and adolescent mental health services [55, 56]. The prevalence of borderline/clinical range symptoms of 30.6% for any psychiatric disorder and 48.6% for mood disorders, were as expected substantially higher in this clinical sample than is reported in the general population (mean YSR Total Problems scores 35.3) [52]. Girls had significantly higher symptom load than boys in all diagnostic groups. It must be taken into account that our sample was a follow-up of former outpatients with a high degree of comorbidity and complex symptom patterns [15, 57]. This is quite different from patients with a specific disorder without comorbidity as recruited to most treatment studies [6, 58]. The participants with the highest symptom scores in our study were girls with mood disorders and those in the group of other psychiatric disorders (e.g., eating disorders, psychotic disorders, autism spectrum disorders). We do not know if the high symptom load in patients with mood disorders was due to persistence of the mood disorder at T_1_, or relapse, but research shows that both persistence rates and relapse rates are high for mood disorders [59]. We have previously reported a high degree of comorbidity after three years among girls in this sample [15], as well as high rates of suicidal ideation and behavior [57], which may contribute to the higher symptom scores compared with boys. Explorative analyses of the T-scores for participants with or without a psychiatric diagnosis at T_2_, showed as expected highest symptom scores among the participants with a diagnosis, and highest scores among girls.

The main feature of the analysis of treatment characteristics was that patients in all diagnostic groups received extensive interventions, as roughly nine out of ten received some type of individual treatment. Disorder specific features were also observed in that those with anxiety and mood disorders at T_1_ had received the highest number of psychotherapy sessions, whereas ADHD and other disorders had the highest rate of medication, both indicating a high disease burden at T_1_. The different treatment methods could furthermore depend on disorder specific features, for example verbal deficits and problems with emotion processing often present with ADHD [60, 61]. Moreover, medication has been long established as an effective treatment for ADHD. When investigating treatment procedures given to the participants in this study, we should also keep in mind that there was a high degree of comorbidity at T_1_, as nearly one out of three had comorbid disorders in addition to their primary disorder.

Treatment characteristics were not found to be analogues for girls and boys. More than one in two girls compared with only about one in four boys received ten or more psychotherapy sessions. Moreover, girls received significantly more psychotherapy sessions than boys in all diagnostic groups. We need to be mindful that the girls in this sample were significantly older than the boys when participating in the study. This may have an impact on the findings related to the use of psychotherapy among girls, because higher age may imply higher maturity to utilize the benefits of psychotherapeutic approaches. The opposite pattern was found for medication, where boys were more likely to receive medication compared with girls. The differences in treatment provided may reflect that more boys than girls had ADHD, for which medication is the treatment of choice. Nonetheless, even when having the same diagnosis of ADHD, there were still some gender differences. Consistent with our results, previous research has found that girls with ADHD are less likely to be prescribed medication unless they have prominent externalizing problems [62].

Positive associations were found between the number of psychotherapy sessions and symptom load for girls only, overall and in the groups of anxiety disorders and ADHD, possibly because these groups had a high and complex symptom pattern in the first place, resulting in longer treatment. Results from the CAMELS study found that despite receiving evidence-based treatments for anxiety, only 22% were in stable remission across all four years when they were assessed, 30% were chronically ill, and 48% experienced relapse [10]. Furthermore, the positive association between psychotherapy sessions and symptom load for girls with ADHD may reflect both the high symptom load for these girls and that fewer girls than boys received medication for this disorder. The positive association between receiving medication at baseline and higher symptom load at follow-up were found only in boys. As a counterintuitive result, this warrants replication in future studies. One might speculate that this could have been due to gender-specific differences in initial diagnoses, less additional psychotherapy in boys, or possibly gender-specific differences in initial symptom load. When performing explorative analyses for the participants with or without a psychiatric diagnosis at T_2_, we found that the associations between the number of psychotherapy sessions and symptom load was only present in the subgroup with a psychiatric diagnosis at T_2_. This fits with the assumption that this is the presumed group with most symptom burden.

Beyond this, no associations were found between treatment characteristics at baseline and symptom load at follow-up, whether for counselling parents nor municipal services. This may be due to the complexities in classifying outpatient treatment, symptom patterns, and comorbidity in this sample. It is challenging to implement high quality and targeted treatment in adolescence, if the burden of comorbid psychiatric disorders is high [13, 14]. There are few transdiagnostic treatment options available today, which could expand treatment benefits beyond what is produced by therapies for any single disorder [12]. One example of a transdiagnostic approach is the Modular Approach to Therapy for Children with Anxiety, Depression, Trauma, or Conduct Problems (MATCH) [19]. Another aspect is that effect sizes for therapies in children and adolescents have been found to be significantly smaller than for adults [11, 63]. We surmise that in our study those with the highest symptom burden at baseline received the most extensive treatment procedures. Therefore, this observational follow-up study is not intended to evaluate effect of the treatment provided, as this would require randomized controlled trial methods. Furthermore, it is important to investigate how to use the resources in CAMHS in the best possible way, for example to find the optimal scope of psychotherapy for adolescent psychiatric patients.

Factors that positively can influence outcomes for adolescent patients are of great interest and importance. The concept of resilience may point to such factors, yielding more positive psychological outcome than would be expected based on risk exposure. The fact that higher self-reported personal and social resources may have a protective potential in relation to adolescents’ symptom load, may be due to a variety of factors including cognitive level. The self-reported resilience factors found in this clinical sample showed a pattern across subscales similar to previous research within a general population [38]. Overall, the levels of resilience factors were fairly low, indicating the vulnerability typical in a clinical sample. This vulnerability may also partly explain the high symptom load after three years [15, 57]. When differentiating by psychiatric disorders, patients with mood disorders had the lowest levels of resilience factors for both genders. We cannot exclude that the presence of a mood disorder, particularly depression, may have had a negative impact on resilience scores reported at the same time, and possibly biased the findings. Consistent with our hypothesis, resilience factors were associated with symptom scores, across all subscales and both genders. We found that higher levels of resilience factors at baseline were linked to lower symptom severity three years later, overall and in all diagnostic groups, except for mood disorders among boys. When performing explorative analyses for the participants with or without a psychiatric diagnosis at T_2_, the findings were present in both groups.

We found gender differences in resilience factors that were similar to results from earlier research [36, 38], especially concerning the subscale Personal Competence. The considerably higher scores for boys in this subscale are consistent with research showing boys to report higher levels than girls on constructs such as general self-esteem and self-efficacy [64]. A large meta-analysis including 85 longitudinal studies [65] concluded that the effect of low self-esteem on depression and anxiety is substantial in the general population, and this association has also been reported in studies with clinical samples [41, 66]. We hypothesized based on previous research that girls would report higher scores than boys on Social Resources [36, 38, 45]. This was not verified as girls reported lower levels for all resilience factors. One reason for the lower scores among girls may be their higher prevalence of mood disorders compared to boys, and that especially depression has affected the self-reported scores among girls [67]. The results for boys were in accordance with previous studies [36, 38, 40, 44, 45] and our hypotheses that they had higher resilience scores than girls in Personal and Social Competences.

Previous studies have investigated interventions promoting resilience in children and adolescents. A recent systematic review and meta-analysis of resilience training programs and interventions shows that interventions based on a combination of mindfulness techniques and CBT seem to have a positive impact on individual resilience [68]. Also, a recent literature review showed that resilience was promoted in children and youth by strengthening home and school environments [69]. This research highlights that resilience can be improved through interventions among children and adolescents.

Strengths of the present study include a large clinical sample receiving standard psychiatric clinical care, with reassessment after three years with a high retention rate. Another strength is that the psychiatric diagnoses at T_1_ represent clinical practice as they were classified by clinicians within a multi-disciplinary team, according to the current diagnostic classification system and based on all available clinical information. The diagnoses were not only based on self-report measures of symptoms. Associations were examined when adjusted for age and SES as possible confounders. Some limitations need to be taken into consideration. At the initial recruitment, the rate of enrollment was less than ideal [15, 70], and this may have biased the results. However, the participants at T_1_ did not differ in age, gender or reason for referral compared to non-participants. We may have lost especially patients with high symptom load and impaired function at baseline, as is typical, especially boys since they were underrepresented among participants. Also, the number of participants was low for some diagnostic groups, requiring us to merge some diagnoses into one larger group, which limited the generalizability of the results for these disorders. Association analyses between resilience and symptom load for boys with mood disorders may have been affected by low numbers and therefore low power. Another limitation is that the assessment of psychiatric disorders of study participants at T_1_ were not done by using the same structured procedure, rather reflecting clinical practice influenced by patient presentation and clinical preferences. Self-report was used to measure symptom load at T_2_ and should ideally be supplemented by clinical interview and proxy report. Although YSR is a widely used and validated instrument, some information bias cannot be excluded when using only self-report. As different informants may have different standards for rating problems, adding proxy reports from parents using the Child Behavior Check List (CBCL) could have balanced the information [51]. Low agreement between self-report (YSR) and parent-report (CBCL) may appear, depending on subjective factors of both respondents [71]. Furthermore, social desirability may lead to self-reported better competences and resources. This study did not have data from YSR available at T_1_, which would have strengthened the study and made it possible to compare resilience scores with symptom load at different times.

### Clinical implications

The results of this study bring an essential message to clinical practice. Despite clinical interventions that were intended to address presenting disorders, the high symptom load reported by girls, and by those with mood disorders, is especially noteworthy. Even though clinicians know about the increase of psychiatric disorders during adolescence, the self-reported high symptom load in this sample of former patients should be an additional reminder. The results point to the importance of focusing on this vulnerable group of patients at the transition from youth to young adulthood. The burden of mental health problems in adolescence must be acknowledged and motivate the search for more effective interventions, either targeted or transdisciplinary. Systematic use of validated screening measures will increase the likelihood that symptoms are properly recognized. Higher reported resilience factors were associated with lower symptom load after three years, suggesting the protective potential of personal resources. Future research needs to expand knowledge on how resilience factors can be developed or enhanced through intervention and whether this leads to reduced symptom load several years later.

## Conclusions

In this clinical sample of adolescents reassessed after three years, one out of three had symptom loads in the borderline/clinical range. Girls had the highest symptom load, especially those with previous mood disorders. Treatment were extensive in form and duration for large portions and nine out of ten had received individual treatment. Self-reported resilience factors appeared lowest among patients with mood disorders and highest among ADHD patients, and lower among girls than boys in all diagnostic groups. Higher self-reported personal and social resources were associated with lower symptom load after three years, suggesting that they can have a protective potential. The results accentuate the importance of continuous research to find the most effective interventions and facilitating factors for adolescents with psychiatric disorders to enhance optimal function.

## Supplementary Information

**Additional file 1: Table S1**. Medication differentiated by primary psychiatric disorders at T_1_. **Table S2**. Treatment procedures at T_1_ differentiated by psychiatric disorders. **Table S3**. Resilience measures at T_1_ differentiated by psychiatric disorders. **Table S4**. Linear regression analysis with YSR Total Problems T-score at 3-year follow up as dependent variable and resilience factors as covariates with all subscales simultaneously. **Table S5**. Linear regression analysis with YSR Total Problems T-score at 3-year follow up as dependent variable and Total READ as covariate, differentiated by psychiatric disorders.

## Data Availability

The datasets analyzed during the current study are not publicly available due to privacy policy, but they are obtainable from the corresponding author on acceptable request.

## Abbreviations

ADHD: Attention Deficit Hyperactivity Disorder
ATC-codes: Anatomical Therapeutic Chemical codes
CAMELS: Child/Adolescent Anxiety Multimodal Extended Long-Term Study
CAMHS: Child and Adolescent Mental Health Services
CBT: Cognitive Behavioral Therapy
CI: Confidence interval
DSM-IV-TR: Diagnostic and Statistical Manual of Mental Disorders IV Text revision
K-SADS: Kiddie SADS: Schedule for Affective Disorders and Schizophrenia for School-Age Children
MATCH: Modular Approach to Therapy for Children with Anxiety, Depression, Trauma, or Conduct Problems
READ: Resilience Scale of Adolescents
RD: Risk difference
SES: Socioeconomic status
YSR: Achenbach System of Empirically Based Assessment - Youth Self Report
