# Supplementary material for: Association of treatment procedures and resilience to symptom load three-years later in a clinical sample of adolescent psychiatric patients
Source: BMC Psychiatry. 2021 Aug 19;21:411. doi: 10.1186/s12888-021-03417-6 (PMC8377856; doi:10.1186/s12888-021-03417-6)
Supplement: Supplementary file 1 — Additional file 1: Table S1. Medication differentiated by primary psychiatric disorders at T1. Table S2. Treatment procedures at T1 differentiated by psychiatric disorders. Table S3. Resilience measures at T1 differentiated by psychiatric disorders. Table S4. Linear regression analysis with YSR Total Problems T-score at 3-year follow up as dependent variable and resilience factors as covariates with all subscales simultaneously. Table S5. Linear regression analysis with YSR Total Problems T-score at 3-year follow up as dependent variable and Total READ as covariate, differentiated by psychiatric disorders. [file 12888_2021_3417_MOESM1_ESM.docx]

**Table S1 - Medication differentiated by primary psychiatric disorders at T_1_, overall and separately for girls and boys**

|  |  |  | **Medication** | | | | | | | | | | | |
| --- | --- | --- | --- | --- | --- | --- | --- | --- | --- | --- | --- | --- | --- | --- |
| **Psychiatric disorders^a^ T_1_** |  |  | **Any medication^b^** | | **Antidepressants^c^** | | **Psycholeptics^d^** | | **Psychostimulants^e^** | | **Antiepileptics^f^** | | **Medication for sleeping problems^g^** | |
|  | n | (%) | n | (%) | n | (%) | n | (%) | n | (%) | n | (%) | n | (%) |
| **Total sample** |  |  |  |  |  |  |  |  |  |  |  |  |  |  |
| Any psych disorder | 445^h^ |  | 236 | (53.0) | 55 | (12.4) | 14 | (3.1) | 166 | (37.3) | 7 | (1.6) | 21 | (4.7) |
| Anxiety disorders | 116 | (26.1) | 34 | (29.3) | 26 | (22.4) | <5^i^ |  | 7^j^ | (6.0) | <5 |  | 5 | (4.3) |
| Mood disorders | 68 | (15.3) | 26 | (38.2) | 19 | (27.9) | <5 |  | <5 |  | <5 |  | 6 | (8.8) |
| ADHD | 172 | (38.7) | 137 | (79.7) | 5 | (2.9) | <5 |  | 133 | (77.3) | <5 |  | <5 |  |
| Other psych disorders | 87 | (19.6) | 39 | (44.8) | 5 | (5.7) | 9 | (10.3) | 24 | (27.6) | <5 |  | 6 | (6.9) |
| **Girls** |  |  |  |  |  |  |  |  |  |  |  |  |  |  |
| Any psych disorder | 253 |  | 114 | (45.1) | 41 | (16.2) | 5 | (2.0) | 64 | (25.3) | 7 | (2.8) | 16 | (6.3) |
| Anxiety disorders | 79 | (31.2) | 22 | (27.8) | 17 | (21.5) | 0 |  | <5 |  | <5 |  | 5 | (6.3) |
| Mood disorders | 56 | (22.1) | 21 | (37.5) | 15 | (26.8) | <5 |  | <5 |  | <5 |  | <5 |  |
| ADHD | 77 | (30.4) | 58 | (75.3) | 5 | (6.5) | 0 |  | 55 | (71.4) | <5 |  | <5 |  |
| Other psych disorders | 40 | (15.8) | 13 | (32.5) | <5 |  | <5 |  | <5 |  | <5 |  | <5 |  |
| **Boys** |  |  |  |  |  |  |  |  |  |  |  |  |  |  |
| Any psych disorder | 192 |  | 122 | (63.5) | 14 | (7.3) | 9 | (4.7) | 102 | (53.1) | 0 |  | 5 | (2.6) |
| Anxiety disorders | 37 | (19.3) | 12 | (32.4) | 9 | (24.3) | <5 |  | <5 |  | 0 |  | 0 |  |
| Mood disorders | 12 | (6.3) | 5 | (41.7) | <5 |  | 0 |  | <5 |  | 0 |  | <5 |  |
| ADHD | 95 | (49.5) | 79 | (83.2) | 0 |  | <5 |  | 78 | (82.1) | 0 |  | 0 |  |
| Other psych disorders | 47 | (24.5) | 26 | (55.3) | <5 |  | 6 | (12.8) | 20 | (42.6) | 0 |  | <5 |  |

**Note:** *^a^ Psychiatric disorder includes* ***only primary diagnoses***

*^b^ Medication includes medication for psychiatric disorders; according to Anatomical Therapeutic Chemical (ATC) codes Yes/No*

*^c^ Antidepressants ATCN06A - B03, B04, B06, B10, X03 and X16*

*^d^ Psycholeptics ATCN05 - AH04, AX08 and AX12*

*^e^ Psychostimulants ATCN06B - A01, A04 and A09*

*^f^ Antiepileptics ATCN03 - AD01 and AX09*

*^g^ Medication for sleeping problems: Psycholeptics ATCN05 - CF01 and CH01, and Antihistamins ATCR06 including AD01*

*^h^ Information about medication was missing from 2 participants*

*^i^ Numbers below 5 are not given*

*^j^ All 7 patients had ADHD as additional diagnosis*

**Table S2 Treatment procedures at T_1_ differentiated by psychiatric disorders, including comorbid disorders at T_1_, comparing girls versus boys**

|  | **Treatment procedures T_1_** | | | | | | | | | | | | | | |
| --- | --- | --- | --- | --- | --- | --- | --- | --- | --- | --- | --- | --- | --- | --- | --- |
|  | **Psychotherapy^b^** | | | | | | |  | **Medication^c^** | | | | | | |
| **Psychiatric disorders^a^ T_1_** | **Girls** | | **Boys** | | **Girls versus Boys** | | |  | **Girls** | | **Boys** | | **Girls versus Boys** | | |
|  | **n** | **(%)** | **n** | **(%)** | **RD (%)** | **95% CI^d^** | ***p^e^*** |  | **n** | **(%)** | **n** | **(%)** | **RD (%)** | **95% CI** | ***p*** |
| **Any psych disorder** | 224/242 | (92.5) | 134/182 | (73.6) | 18.9 | 11.2 to 26.4 | <0.001 |  | 114/253 | (45.1) | 122/192 | (63.5) | -18.4 | -27.3 to -9.1 | <0.001 |
| **Anxiety disorders** | 93/96 | (96.9) | 40/46 | (87.0) | 9.9 | 1.0 to 22.7 | 0.023 |  | 36/102 | (35.3) | 20/48 | (41.7) | -6.4 | -22.8 to 9.7 | 0.452 |
| **Mood disorders** | 81/83 | (97.6) | 15/18 | (83.3) | 14.3 | 1.9 to 36.9 | 0.011 |  | 44/88 | (50.0) | 8/18 | (44.4) | 5.6 | -18.6 to 27.9 | 0.667 |
| **ADHD** | 73/84 | (86.9) | 77/112 | (68.7) | 18.2 | 6.3 to 28.8 | 0.003 |  | 66/87 | (75.9) | 102/120 | (85.0) | -9.1 | -20.4 to 1.6 | 0.097 |
| **Other psych disorders** | 43/44 | (97.7) | 36/49 | (73.4) | 24.3 | 10.2 to 38.1 | 0.001 |  | 16/49 | (32.7) | 30/50 | (60.0) | -23.3 | -40.6 to -3.6 | 0.006 |

**Note:** *RD = Risk Difference, CI = Confidence Interval, p = p-value*

*^a^ Psychiatric disorders include both primary and additional diagnoses*

*^b^ Psychotherapy includes both specified and unspecified psychotherapy, measured by Yes/No*

*^c^ Medication includes medication for psychiatric disorders; according to Anatomical Therapeutic Chemical (ATC) codes Yes/No*

*^d^ Newcomb hybrid score*

*^e^ Pearson Chi squared test*

**Table S3 Resilience measures at T_1_ differentiated by psychiatric disorders, including comorbid disorders at T_1_, comparing girls versus boys**

|  | **Resilience measures - Girls versus Boys** | | | | | | | | | | | | | | | | | | | | | | | | |
| --- | --- | --- | --- | --- | --- | --- | --- | --- | --- | --- | --- | --- | --- | --- | --- | --- | --- | --- | --- | --- | --- | --- | --- | --- | --- |
| **Psychiatric**  **disorders^a^ T_1_** | **Personal competence** | | | **Social competence** | | | | | **Structured style** | | | **Family cohesion** | | | | | **Social resources** | | | **Total READ** | | | |  |  |
|  | **Diff** | **95% CI** | ***p*** | | **Diff** | **95% CI** | ***p*** | **Diff** | | **95% CI** | ***p*** | | **Diff** | **95% CI** | ***p*** | **Diff** | | **95% CI** | ***p*** | | **Diff** | **95% CI** | ***p*** | |  |
| **Any psychiatric disorder** | -0.8 | -1.0 to -0.7 | <0.001 | | -0.3 | -0.5 to -0.1 | 0.001 | -0.5 | | -0.7 to -0.4 | <0.001 | | -0.4 | -0.6 to -0.3 | <0.001 | -0.3 | | -0.4 to -0.1 | <0.001 | | -0.5 | -0.6 to -0.3 | <0.001 | |  |
| **Anxiety disorders** | -0.7 | -1.0 to -0.5 | <0.001 | | -0.5 | -0.8 to -0.2 | 0.003 | -0.4 | | -0.7 to -0.1 | 0.014 | | -0.5 | -0.8 to -0.2 | 0.003 | -0.3 | | -0.6 to -0.0 | 0.029 | | -0.5 | -0.7 to -0.2 | <0.001 | |  |
| **Mood disorders** | -0.6 | -1.0 to -0.2 | 0.007 | | -0.2 | -0.6 to 0.3 | 0.388 | -0.2 | | -0.6 to 0.2 | 0.361 | | -0.2 | -0.7 to 0.3 | 0.405 | -0.3 | | -0.8 to 0.1 | 0.146 | | -0.3 | -0.6 to 0.0 | 0.080 | |  |
| **ADHD** | -0.7 | -1.0 to -0.5 | <0.001 | | -0.3 | -0.5 to 0.0 | 0.057 | -0.7 | | -0.9 to -0.4 | <0.001 | | -0.4 | -0.6 to -0.1 | 0.002 | -0.2 | | -0.4 to -0.0 | 0.022 | | -0.5 | -0.7 to -0.3 | <0.001 | |  |
| **Other psychiatric disorders** | -0.8 | -1.1 to -0.5 | <0.001 | | -0.2 | -0.6 to 0.2 | 0.335 | -0.4 | | -0.8 to -0.1 | 0.019 | | -0.3 | -0.6 to 0.2 | 0.227 |  | | -0.4 to 0.2 | 0.587 | | -0.4 | -0.6 to -0.1 | 0.013 | |  |

**Note:** *Resilience measures using READ = Resilience Scale for Adolescents,* *based on a 5-point Likert scale (1 = Totally Disagree to 5 = Totally Agree, higher scores indicate higher level of resilience factors), Diff = Difference Girls versus Boys, CI = Confidence Interval, p = p-value*

*^a^ Psychiatric disorders include both primary and additional diagnoses*

**Table S4 Linear regression analysis with YSR Total Problems T-score at 3-year follow up as dependent variable and resilience factors as covariates with all subscales simultaneously, adjusted for age and SES, overall and separately for girls and boys**

|  |  | **YSR Total Problems T-score at T_2_** | | |
| --- | --- | --- | --- | --- |
| **Resilience measures** |  | **Adjusted for age T_1_ and SES** | | |
|  | **n** | **β** | **95% CI** | ***p*-value** |
| **Total sample** | 447 |  |  |  |
| Personal competence | 446/447 | -11.4 | -16.1 to -6.7 | <0.001 |
| Social competence | 444/447 | 2.9 | -1.0 to 6.7 | 0.146 |
| Structured style | 445/447 | -0.7 | -5.0 to 3.6 | 0.759 |
| Family cohesion | 444/447 | -4.8 | -9.1 to -0.6 | 0.026 |
| Social resources | 444/447 | 0.1 | -5.0 to 5.2 | 0.960 |
| **Girls** | 254 |  |  |  |
| Personal competence | 254/254 | -8.6 | -15.7 to -1.4 | 0.019 |
| Social competence | 253/254 | 2.8 | -2.6 to 8.1 | 0.307 |
| Structured style | 254/254 | 0.4 | -6.2 to 7.1 | 0.897 |
| Family cohesion | 253/254 | -7.6 | -13.2 to -1.9 | 0.009 |
| Social resources | 253/254 | 0.3 | -6.3 to 6.9 | 0.929 |
| **Boys** | 193 |  |  |  |
| Personal competence | 192/193 | -6.8 | -14.1 to 0.5 | 0.067 |
| Social competence | 191/193 | 0.5 | -5.3 to 6.2 | 0.873 |
| Structured style | 191/193 | -2.9 | -8.1 to 2.3 | 0.268 |
| Family cohesion | 191/193 | -0.6 | -7.1 to 5.9 | 0.859 |
| Social resources | 191/193 | -0.8 | -8.9 to 7.3 | 0.849 |

**Note:** *Symptom load is measured by using Youth Self Report (YSR, Achenbach System of Empirically Based Assessment), Total Problem T-score, Resilience measures using READ = Resilience Scale for Adolescents,* *based on a 5-point Likert scale (1 = Totally Disagree, 5 = Totally Agree, higher scores indicate higher level of resilience factors), SES = Socioeconomic Status measured by level of mothers education (1 = lowest level of education, 9 = highest level of education), β = Regression Coefficient, CI = Confidence Interval*

**Table S5 Linear regression analysis with YSR Total Problems T-score at 3-year follow up as dependent variable and Total READ as covariate, adjusted for age and SES, differentiated by psychiatric disorders**

|  |  |  |  |  |  | **YSR Total Problems T-score at T_2_** | | | | | |
| --- | --- | --- | --- | --- | --- | --- | --- | --- | --- | --- | --- |
| **Psychiatric disorders T_1_^a^** |  |  | **Total READ** | |  | **Adjusted for age T_1_ and SES** | | | | | |
|  | **n** | **(%)** | **n** | **%** |  | **n** | **%** | | **β** | **95% CI** | ***p*-value** |
| **Total sample** |  |  |  |  |  |  |  |  | |  |  |
| Any psychiatric disorder | 447 |  | 444/447 | (99.3) |  | 326/444 | (73.4) | -15.3 | | -18.8 to -11.9 | <0.001 |
| Anxiety disorders | 151/447 | (33.8) | 151/151 | (100) |  | 106/151 | (70.2) | -16.4 | | -21.5 to -11.2 | <0.001 |
| Mood disorders | 107/447 | (23.9) | 106/107 | (99.1) |  | 68/106 | (64.2) | -23.3 | | -33.0 to -13.5 | <0.001 |
| ADHD | 207/447 | (46.3) | 205/207 | (99.0) |  | 159/205 | (77.6) | -13.2 | | -18.1 to -8.2 | <0.001 |
| Other psychiatric disorder | 99/447 | (22.1) | 98/99 | (99.0) |  | 77/98 | (78.6) | -18.5 | | -27.2 to -9.8 | <0.001 |
| **Girls** |  |  |  |  |  |  |  |  | |  |  |
| Any psychiatric disorder | 254 |  | 253/254 | (99.6) |  | 180/253 | (71.1) | -14.2 | | -19.1 to -9.2 | <0.001 |
| Anxiety disorders | 103/254 | (40.6) | 103/103 | (100) |  | 68/103 | (66.0) | -15.7 | | -22.4 to -9.0 | <0.001 |
| Mood disorders | 88/254 | (34.7) | 88/88 | (100) |  | 58/88 | (65.9) | -20.8 | | -31.3 to -10.3 | <0.001 |
| ADHD | 87/254 | (34.3) | 86/87 | (98.8) |  | 66/86 | (76.7) | -11.7 | | -20.3 to -3.2 | 0.008 |
| Other psychiatric disorder | 49/254 | (19.3) | 49/49 | (100) |  | 39/49 | (79.6) | -15.9 | | -30.4 to -1.4 | 0.033 |
| **Boys** |  |  |  |  |  |  |  |  | |  |  |
| Any psychiatric disorder | 193 |  | 191/193 | (99.0) |  | 146/191 | (76.4) | -11.4 | | -16.2 to -6.6 | <0.001 |
| Anxiety disorders | 48/193 | (24.9) | 48/48 | (100) |  | 38/48 | (79.2) | -15.3 | | -26.6 to -4.1 | 0.009 |
| Mood disorders | 19/193 | (9.8) | 18/19 | (94.7) |  | 10/18 | (55.6) | -9.1 | | -33.6 to 15.4 | 0.398 |
| ADHD | 120/193 | (62.2) | 119/120 | (99.2) |  | 93/119 | (78.2) | -10.6 | | -16.7 to -4.5 | 0.001 |
| Other psychiatric disorder | 50/193 | (25.9) | 49/50 | (98.0) |  | 38/49 | (77.6) | -15.0 | | -23.9 to -6.0 | 0.002 |

**Note:** *Symptom load is measured by using Youth Self Report (YSR, Achenbach System of Empirically Based Assessment), Total Problem T-score, Resilience measures using READ = Resilience Scale for Adolescents,* *based on a 5-point Likert scale (1 = Totally Disagree, 5 = Totally Agree, higher scores indicate higher level of resilience factors), SES = Socioeconomic Status measured by level of mothers education (1 = lowest level of education, 9 = highest level of education), β = Regression Coefficient, CI = Confidence Interval*

*^a^ Psychiatric disorders include both primary and additional diagnoses*
